# Amphiregulin acts as an autocrine growth factor in two human polarizing colon cancer lines that exhibit domain selective EGF receptor mitogenesis

**DOI:** 10.1038/sj.bjc.6690456

**Published:** 1999-06

**Authors:** L Damstrup, S K Kuwada, P J Dempsey, C L Brown, C J Hawkey, H S Poulsen, H S Wiley, R J Coffey

**Affiliations:** Departments of Medicine and Cell Biology, Vanderbilt University School of Medicine and Veterans Affairs Medical Center, Nashville, TN 37232, USA; Section for Radiation Biology, University Hospital Copenhagen, Finsen Center 3993, Copenhagen DK-2100, Denmark; Departments of Medicine and Pathologyand Veterans Affairs Medical Center, University of Utah, Salt Lake City, UT 84132, USA

**Keywords:** EGFR, polarized cells, colon cancer cell lines, ligand and proliferation

## Abstract

Colonic enterocytes, like many epithelial cells in vivo, are polarized with functionally distinct apical and basolateral membrane domains. The aims of this study were to characterize the endogenous epidermal growth factor (EGF)-like ligands expressed in two polarizing colon cancer cell lines, HCA-7 Colony 29 (HCA-7) and Caco-2, and to examine the effects of cell polarity on EGF receptor-mediated mitogenesis. HCA-7 and Caco-2 cells were grown on plastic, or as a polarized monolayer on Transwell filters. Cell proliferation was measured by ^3^H-thymidine incorporation and EGF receptor (EGFR) binding was assessed by Scatchard analysis. EGFR ligand expression was determined by Northern blot analysis, reverse transcription polymerase chain reaction, metabolic labelling and confocal microscopy. We found that amphiregulin (AR) was the most abundant EGFR ligand expressed in HCA-7 and Caco-2 cells. AR was localized to the basolateral surface and detected in basolateral-conditioned medium. Basolateral administration of neutralizing AR antibodies significantly reduced basal DNA replication. A single class of high-affinity EGFRs was detected in the basolateral compartment, whereas the apical compartment of polarized cells, and cells cultured on plastic, displayed two classes of receptor affinity. Basolateral administration of transforming growth factor alpha (TGF-α) or an EGFR neutralizing antibody also resulted in a dose-dependent stimulation or attenuation, respectively, of DNA replication. However, no mitogenic response was observed when these agents were added to the apical compartment or to confluent cells cultured on plastic. We conclude that amphiregulin acts as an autocrine growth factor in HCA-7 and Caco-2 cells, and EGFR ligand-induced proliferation is influenced by cellular polarity. © 1999 Cancer Research Campaign


*British Journal of Cancer* (1999) **80**(7), 1012–1019
© 1999 Cancer Research Campaign
Article no. bjoc.1998.0456

### Amphiregulin acts as an autocrine growth factor in two human polarizing colon cancer lines that exhibit domain selective EGF receptor mitogenesis

**L Damstrup[1,2], SK Kuwada[3,4], PJ Dempsey[1], CL Brown[1], CJ Hawkey[1], HS Poulsen[2], HS Wiley[3] and RJ Coffey Jr[1]**

[1]Departments of Medicine and Cell Biology, Vanderbilt University School of Medicine and Veterans Affairs Medical Center, Nashville, TN 37232, USA; [2]Section for Radiation Biology, University Hospital Copenhagen, Finsen Center 3993, Copenhagen DK-2100, Denmark; [3]Departments of Medicine and Pathology, [4]and Veterans Affairs Medical Center, University of Utah, Salt Lake City, UT 84132, USA

**Summary** Colonic enterocytes, like many epithelial cells in vivo, are polarized with functionally distinct apical and basolateral membrane domains. The aims of this study were to characterize the endogenous epidermal growth factor (EGF)-like ligands expressed in two polarizing colon cancer cell lines, HCA-7 Colony 29 (HCA-7) and Caco-2, and to examine the effects of cell polarity on EGF receptor-mediated mitogenesis. HCA-7 and Caco-2 cells were grown on plastic, or as a polarized monolayer on Transwell filters. Cell proliferation was measured by $^{3}$H-thymidine incorporation and EGF receptor (EGFR) binding was assessed by Scatchard analysis. EGFR ligand expression was determined by Northern blot analysis, reverse transcription polymerase chain reaction, metabolic labelling and confocal microscopy. We found that amphiregulin (AR) was the most abundant EGFR ligand expressed in HCA-7 and Caco-2 cells. AR was localized to the basolateral surface and detected in basolateral-conditioned medium. Basolateral administration of neutralizing AR antibodies significantly reduced basal DNA replication. A single class of high-affinity EGFRs was detected in the basolateral compartment, whereas the apical compartment of polarized cells, and cells cultured on plastic, displayed two classes of receptor affinity. Basolateral administration of transforming growth factor alpha (TGF-α) or an EGFR neutralizing antibody also resulted in a dose-dependent stimulation or attenuation, respectively, of DNA replication. However, no mitogenic response was observed when these agents were added to the apical compartment or to confluent cells cultured on plastic. We conclude that amphiregulin acts as an autocrine growth factor in HCA-7 and Caco-2 cells, and EGFR ligand-induced proliferation is influenced by cellular polarity.

**Keywords:** EGFR; polarized cells; colon cancer cell lines; ligand and proliferation

Six mammalian ligands have been identified that bind to the epidermal growth factor receptor (EGFR): EGF, transforming growth factor-α (TGF-α), amphiregulin (AR), heparin-binding EGF-like growth factor (HB-EGF), betacellulin (BTC) and epiregulin. These ligands have been shown to mediate various cellular functions, including proliferation, differentiation and migration (Basson et al, 1992, 1994; Amemiya et al, 1994; Chen et al, 1994; Traverse et al, 1994). In epithelial cells cultured as a flat monolayer on plastic, it has been shown that TGF-α and AR can act in an autocrine manner (Culouscou et al, 1992; Johnson et al, 1992; Li et al, 1992; Ziober et al, 1993; Qi et al, 1994). Many epithelial cells in vivo, however, are polarized with spatially restricted apical and basolateral compartments. Certain epithelial cell types, most notably non-transformed canine MDCK-II cells, can be cultured in vitro as a polarized monolayer on semipermeable supports (Transwell filters). It has been shown that EGFRs are restricted to the basolateral compartment of these polarized MDCK cells (Maratos-Flier et al, 1987; Dempsey et al, 1994; Hobert et al, 1995) and it is predicted that basolateral, but not apical, administration of EGFR ligands would elicit biological effects.

*Received 3 September 1998*
*Accepted 15 December 1998*

*Correspondence to:* L Damstrup, Section for Radiation Biology, University Hospital Copenhagen, Finsen Center, 3993, 9, Blegdamsvej, Copenhagen DK-2100, Denmark

The present studies were undertaken to examine EGFR-mediated proliferation in two well-differentiated human colon cancer lines, HCA-7 Colony 29 (HCA-7) and Caco-2, that retain the ability to polarize under their appropriate culture conditions (Kirkland 1985; LeBivic et al, 1991; Coffey et al, 1997). Like MDCK cells, Caco-2 cells have been reported to have EGFRs localized predominantly to the basolateral surface (Bishop et al, 1994). We have compared DNA replication in HCA-7 and Caco-2 cells when cultured as a flat monolayer on plastic, and as a polarized monolayer on Transwell filters. As predicted, TGF-α stimulates mitogenesis when added to the basolateral compartment, but not to the apical compartment or to confluent cells cultured on plastic. Differences in expression of high-affinity EGFRs on different cell substrates used to culture the cells may account for these variations. In addition, conclusive evidence for AR autocrine growth activity is presented for these polarized cells. These results emphasize the biological relevance of studying epithelial cells in vitro in a polarized context.

### MATERIALS AND METHODS

### Reagents and antibodies

All cell culture reagents were purchased from Gibco Laboratories (Grand Island, NY, USA) and all chemicals were purchased from Sigma Chemical Co. (St Louis, MO, USA), unless otherwise indicated. [$^{35}$S]-Translabel was purchased from ICN Radiochemical (Irvine, CA, USA). Protein A-agarose and Iodo-beads were purchased from Pierce Chemical Co. (Rockford, IL, USA). All electrophoresis reagents were purchased from Bio-Rad Laboratories (Hercules, CA, USA). [$^{3}$H]-thymidine was purchased from DuPont (Boston, MA, USA). Rainbow markers and $^{125}$Iodine were purchased from Amersham (Arlington Heights, IL, USA).

Recombinant human TGF-α was a gift from Triton BioScience (Alameda, CA, USA). Recombinant human AR and monoclonal antibodies to human AR (AR 6R1C2.4, AR 18.4.6, AR 12.38.4 and AR 4.14.18) were generously provided by Dr Greg Plowman and Barbara Thorne (Bristol-Myers Squibb Pharmaceutical Research Institute, Seattle, WA, USA). Monoclonal antibody to human EGFR (mAb 528) was generously provided by Dr Hideo Masui (Memorial Sloan-Ketting Cancer Center, NY, USA). Affinity-purified rabbit antisera to mouse immunoglobulin was purchased from Cappel Laboratories (Durham, NC, USA). Vectashield mounting medium was purchased from Vector Laboratories (Burlingame, CA, USA). Normal donkey serum and CY3-conjugated donkey anti-mouse IgG were purchased from Jackson ImmunoResearch Laboratories (West Grove, PA, USA).

### Cells and cell culture

HCA-7 cells were obtained from Dr Susan Kirkland (ICRF, London, UK) and used between passage 20 and 35. Caco-2 cells were obtained from Dr E Rodriguez-Boulan (Cornell University Medical College, New York, NY, USA) and used between passage 90 and 100. Both cell lines were grown in Dulbecco's minimum essential medium (DMEM) supplemented with 10% fetal bovine serum (Intergen, Purchase, NY, USA), as previously described (12). When cultured on Transwell filters (pore size 0.4 μM; Costar, Cambridge, MA, USA), cells were seeded at $1.5 \times 10^7$, $1.5 \times 10^6$, $4 \times 10^5$ cells on 75-, 24.5- and 12-mm Transwell filters, respectively, unless otherwise stated. Confluence was obtained 24 h after seeding cells at these concentrations.

To evaluate the fidelity of tight junctions, transepithelial electrical resistance across the Transwell filter was measured using a Millicell Electrical Resistance System (Millipore Corp., Bedford, MA, USA). All experiments involving HCA-7 and Caco-2 cells cultured on Transwell filters were performed 4–10 days post-confluence when the resistance was > 400 $\Omega$ cm$^2$.

### Mitogenic assays

For experiments performed on HCA-7 and Caco-2 cells cultured as a monolayer on plastic, cells were seeded in 24-well plates in serum-containing medium and allowed to reach confluence, at which time cells were placed in serum-free medium. After 48 h, the cells were incubated with TGF-α or mAb 528. Cells were treated for 21 h before 1.0 μCi [$^{3}$H]-thymidine was added to the medium and incubated for 3 h (between 21 and 24 h). Cells were washed three times in ice-cold 10% trichloracetic acid (TCA). Cells were then dried and solubilized in 0.1 M sodium hydroxide containing 40 μg ml$^{-1}$ salmon sperm DNA, and an aliquot was counted in a scintillation counter. For experiments performed on HCA-7 and Caco-2 cells grown as a polarized monolayer, cells cultured on Transwell filters for 7–10 days post-confluence were serum-starved for 48 h before TGF-α or mAb 528 was added to either the apical, basal, or both, compartments and treated as described above. For AR antibody neutralization studies, a cocktail of AR monoclonal antibodies (AR 6R1C2.4 was used at 2 μg ml$^{-1}$ and AR 18.4.6, AR 12.38.4, AR 4.14.18 were used at 10 μg ml$^{-1}$) was added to the basal compartment and treated as described above. Mouse monoclonal antibodies UPC-10 and/or MOPC-21 were used as control antibodies.

Results from the proliferation experiments are expressed as fold-change in treated cells over untreated cells (B/Bo). All experiments were performed in triplicate or quadruplicate and repeated at least twice. Student's *t*-test was used to compare the mean of untreated to treated and was regarded as significant if $P \leq 0.05$.

### Isolation of poly-adenosine mRNA and Northern blot analysis

HCA-7 and Caco-2 cells were cultured as a monolayer on plastic and poly-adenosine (poly-A) mRNA was isolated from cells 24 h post-confluence, as described previously (Dobner et al, 1981). For polarized cells grown on Transwell filters, mRNA was isolated 10 days post-confluence. Oligo (dT)-selected mRNA was separated on a 1.0% agarose-formaldehyde gel and transferred to supported Nitroplus nitrocellulose filters (MSI, Westboro, MA, USA), as described previously (Melton et al, 1984). Hybridizations with human probes labelled by RNA polymerase-directed reverse transcription or cDNA insert labelling were performed, as described elsewhere (Melton et al, 1984). As a control for loading and transfer, blots were probed with cyclophillin (1B15) (Danielson et al, 1988).

### Detection of mRNA by RT-PCR

For reverse transcription polymerase chain reaction (RT-PCR), mRNA was isolated from exponentially growing Caco-2 cells, using Quick prep mRNA kit. From this, cDNA was synthesized from 0.5 μg mRNA using the cDNA synthesis kit. PCR was performed using Thermoprime Plus, and specific primers. Specific primers (in 5′–3′ direction) for:

| | |
|---|---|
| TGF-α: | GGGTATTGTGTTGGCTGCGTGC and |
| | GCAAGCGGTTCTTCCCTTCAGG |
| EGF: | TCTGAATGTCCCCTGTCCCACG and |
| | CTGCGACTCCTCACATCTCTGC |
| AR: | TTGAACAGGTAGTTAAGCCCCC and |
| | GGACCGACTCATCATTTATGGC |
| HB-EGF: | CCTTTTGAGAGTCACTTTATCC and |
| | CATTTTCCACATCATAACCTCC |
| BTC: | CCAGAAGTCCTGAAACTAATGG and |
| | TCAAATGAGCAAGGCACTTTGC |
| GAPDH: | AACGGATTTGGTCGTATTGGGC and |
| | TAAGCAGTTGGTGGTGCAGG |

GAPDH was used as a positive and reaction control. All reactions were subjected to 35 cycles of PCR amplification. Each cycle consisted of 30 s of denaturation at 94°C, 1 min at primer specific annealing temperature (70°C for EGF, 66°C for TGF-α and AR, 64°C for BTC, 62°C for HB-EGF and 61°C for GAPDH), and 1 min of primer extension at 72°C. The PCR products were visualized after electrophoresis on a 1% agarose gel containing ethidium bromide. The appearance of specific bands (TGF-α, 421 bp; EGF, 326 bp; AR, 520 bp; HB-EGF, 380; β-cellulin, 489 bp; GAPDH, 446 bp) was evaluated under ultraviolet light and photographed.

*© 1999 Cancer Research Campaign*

### Immunofluorescence and laser scanning confocal microscopy

Immunofluorescent staining of HCA-7 and Caco-2 cells cultured on 12-mm Transwell filters was performed as previously described (Dempsey et al, 1994) with some modifications. Cells were seeded at $1 \times 10^5$ cells per well, and were stained days 4 and 8 post-seeding for Caco-2 and HCA-7 cells, respectively. All staining procedures were performed on ice or at 4°C, unless otherwise stated. For AR staining, cells were paraformaldehyde-fixed and stained with 2 μg ml$^{-1}$ mAb AR 6RIC2.4. Specificity of AR staining was confirmed by preincubating this antibody overnight with 100 ng ml$^{-1}$ recombinant AR, prior to staining. Monoclonal antibody UPC-10 was used as a control.

Laser scanning confocal microscopy was performed using a Zeiss LSM4 confocal microscope (Zeiss, Thornwood, NY, USA). Spatial localization was visualized by z-scanning the images.

### Metabolic labelling

For metabolic labelling experiments, HCA-7 and Caco-2 cells were seeded at $3.75 \times 10^5$ cells per well on 24.5-mm Transwell filters. Prior to labelling, cells were washed two times with serum-free, cysteine–methionine-free DMEM. Then, 2.5 ml of labelling medium (cysteine–methionine-free DMEM with 5% dialysed fetal calf serum (FCS) and 100 μCi ml$^{-1}$ [$^{35}$S]-Translabel) was added to the basal compartment. The apical compartment received 1.5 ml of the same medium lacking [$^{35}$S]-Translabel and the cells were incubated for 16–20 h, or for 2 h and chased for 2 h in medium containing ten-fold excess of cysteine and methionine at 37°C. Caco-2 cells and HCA-7 cells were labelled on days 5 and 8 post-seeding, respectively.

### AR immunoprecipitation from conditioned medium

After metabolic labelling, the apical and basolateral conditioned medium was collected and phenylmethylsulphonyl fluoride was added to a final concentration of 2 μM. The medium was precleared and filtered through a 0.2 μM filter. AR was immunoprecipitated by addition of AR mAb (AR 6R1C2.4, 0.05 μg ml$^{-1}$) overnight, followed by rabbit anti-mouse IgG and 50 μl of a 50% slurry of protein A-agarose beads. The agarose beads were pelleted and washed extensively before being analysed under reducing conditions on a 12.5% sodium dodecyl sulphate poly-acrylamide gel electrophoresis (SDS-PAGE). The gels were fixed, treated with Amplify (Amersham Corp., Arlington Heights, IL, USA) for 30 min and dried. Fluorography was performed using Biomax MR film (Eastman Kodak Co., Rochester, NY, USA).

### Radioactive labelling of EGF

$^{125}$Iodine was used to label 10 μg of EGF using Iodo-beads per manufacturer's recommendations. Unincorporated $^{125}$Iodine was removed with a G-10 Sephadex column equilibrated with phosphate-buffered saline. The specific activity of [$^{125}$I]-labelled EGF was typically between 200 and 350 cpm pg$^{-1}$.

### EGFR binding studies

All procedures were performed at 4°C, unless otherwise stated. The medium was replaced with DMEM containing 20 mM HEPES, pH 7.4 and 0.1% bovine serum albumin (DHB) for approximately 30 min. This medium was removed and cells were washed twice with ice-cold saline, followed by the addition of serial dilutions of [$^{125}$I]-labelled EGF (from 0.5 to 300 ng ml$^{-1}$) prepared in DHB with and without 10 μg EGF to determine non-specific and total EGF binding respectively. The cells were incubated at 0°C to equilibrium (3 h). Unbound [$^{125}$I]-labelled EGF was removed and cells were rinsed five times. Receptor-associated ligand was eluted by adding 1 ml 50 mM glycine–hydrochloric acid, 100 mM sodium chloride, 2 mg ml$^{-1}$ PVP and 2 M urea (pH 3.0) to the cells for 2 min. Eluted material was counted in a γ-counter. Cell numbers were determined with a Coulter counter after trypsinization of parallel sets of plates. The total and non-specific binding were used to calculate bound and bound/free radioligand. The resulting data were analysed by the method of Scatchard (Scatchard, 1949), assuming either one or two receptor

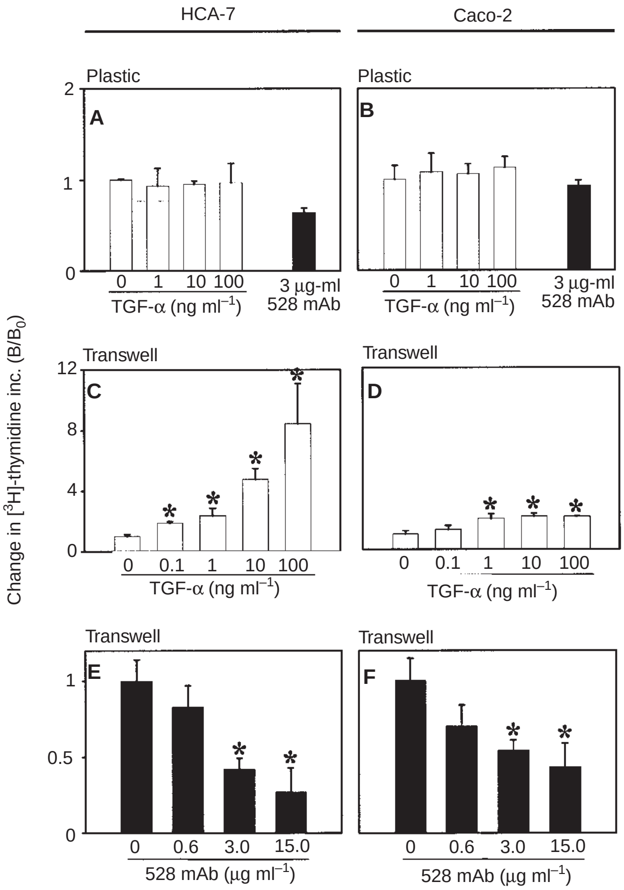

**Figure 1** Alterations in DNA synthesis in HCA-7 and Caco-2 cells cultured as a confluent monolayer on plastic or as a polarized monolayer on Transwell filters. HCA-7 and Caco-2 cells seeded on plastic in 24-well plates were cultured until they became confluent and then placed in serum-free DMEM for 48 h. Polarized cells cultured on Transwell filters (7–10 days post-confluence) were serum starved for 48 h. TGF-α or mAb 528 was administered to the cells for 21 h and [$^3$H]-thymidine labelling was performed as described in Materials and Methods. HCA-7 (**A**) and Caco-2 (**B**) cells cultured as confluent monolayers on plastic were treated with increasing concentrations of TGF-α or mAb528 (3 μg ml$^{-1}$). HCA-7 (**C** and **E**) and Caco-2 (**D** and **F**) cells grown as polarized monolayers on Transwell filters were stimulated with the indicated concentrations of TGF-α or mAb 528 administered to both compartments simultaneously. Vertical bars represent standard deviations. *: significant alteration in mitogenic effect compared to baseline values ($P < 0.05$). Open bars = TGF-α, closed bars = mAb 528

© 1999 Cancer Research Campaign

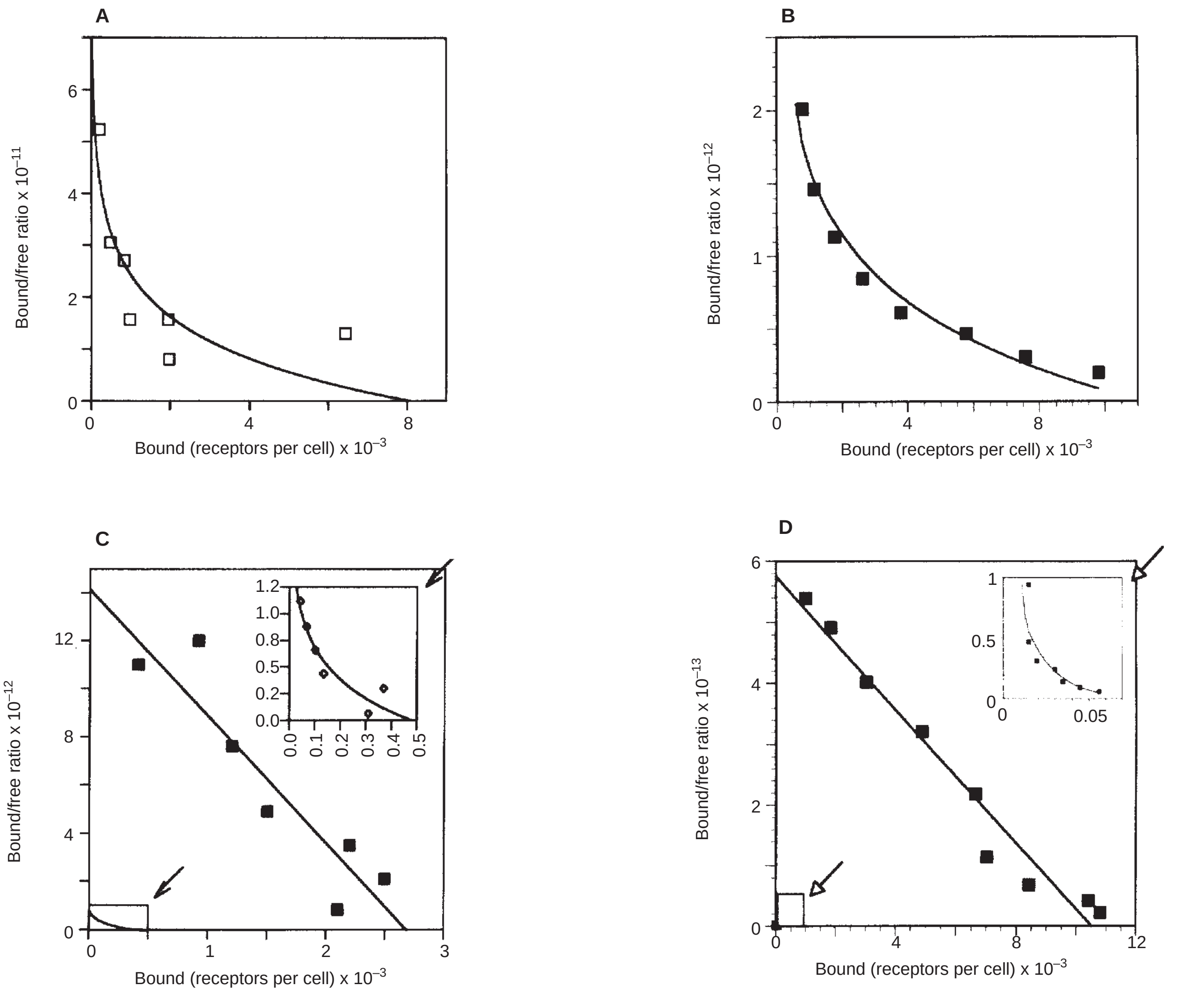

**Figure 2** Binding of [$^{125}$I]-EGF to HCA-7 and Caco-2 cells. HCA-7 (**A**) and Caco-2 (**B**) cells were cultured as a confluent monolayer on plastic, or HCA-7 (**C**) and Caco-2 (**D**) cells were grown as a polarized monolayer on Transwell filters. [$^{125}$I]-labelled EGF binding was performed as described in Materials and Methods. For both HCA-7 and Caco-2 cells cultured as a confluent monolayer on plastic, the curvilinear plots were resolved into two compartments: a high-affinity EGFR with a $K_{D1}$ of 5.0 nM (**A**) and 3.0 nM (**B**) and a low-affinity EGFR with a $K_{D2}$ of 83 nM and 13 nM, respectively. Scatchard analysis of the EGF binding to the basolateral EGFRs demonstrated a linear plot with a single class of high-affinity EGFRs in HCA-7 (**C**) and Caco-2 (**D**) cells ($K_D$ = 2.1 nM and 1.0 nM, respectively). In **C** and **D**, arrow and inset indicate the binding data from apical EGFRs species. Parameters were estimated using non-linear regression by the Levenberg–Marquardt algorithm. Equations used for parameter fitting were from Feldman (Feldman, 1972) and were used with the program Profit (Quantum Soft, Zurich) for the Macintosh computer.

### RESULTS

### Alterations in DNA synthesis after administration of TGFα or mAb 528

In preliminary experiments, we determined that EGFR immunoreactivity was restricted to the basolateral surface of polarized HCA-7 and Caco-2 cells (data not shown). The basolateral compartmentalization of the EGFR led us to examine the mitogenic effects of stimulation and blockade of this receptor under different growth conditions. We first examined the mitogenic effects of TGF-α or mAb 528 added to HCA-7 and Caco-2 cells cultured as a confluent monolayer on plastic. Cell monolayers were maintained serum-free for 48 h before addition of TGF-α (1–100 ng ml$^{-1}$) or mAb 528 (3 μg ml$^{-1}$) for 21 h, followed by a 3 h pulse of [$^{3}$H]-thymidine. In both cell lines, no significant alterations in DNA synthesis were observed after addition of either agent (Figure 1A,B).

The mitogenic effects of stimulation or inhibition of the EGFR in polarized HCA-7 and Caco-2 cells were then examined. Cells cultured for 7–10 days post-confluence on Transwell filters in order to polarize and serum-starved for 48 h were treated as described above. No significant mitogenic effects were seen with the addition of these agents to the apical medium of polarized HCA-7 and Caco-2 cells (data not shown). In contrast, there was a dose-dependent increase in DNA replication after addition of TGF-α to the basolateral medium; 100 ng ml$^{-1}$ TGF-α stimulated [$^{3}$H]-thymidine incorporation 8.4-fold in HCA-7 cells (Figure 1C; $P$ = 0.013) and 3.0-fold in Caco-2 cells (Figure 1D; $P$ = 0.007). We

© 1999 Cancer Research Campaign

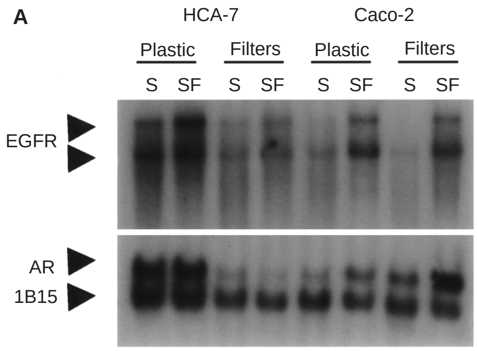

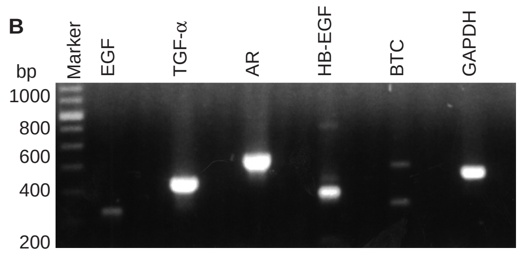

**Figure 3** Steady-state mRNA for EGF-related peptides and EGFR in HCA-7 and Caco-2 cells (**A**). Poly-A mRNA was isolated from HCA-7 and Caco-2 cells cultured either as confluent monolayer on plastic or as a polarized monolayer on Transwell filters under serum-free or serum conditions. Blots were probed with [$^{32}$P]-labelled EGFR cDNA (top panel) or [$^{32}$P]-labelled AR cDNA (middle panel). Arrows indicate the 10 and 6 kb EGFR and the 1.4 kb AR transcripts. Blots were probed with 1B15 to control loading and transfer (bottom panel). In (**B**) cDNA was generated from exponentially growing Caco-2 cells and, using specific primers transcripts for EGF, AR, TGF-α, HB-EGF and BTC was examined also found a dose-dependent decrease in DNA replication following administration of the EGFR neutralizing antibody mAb 528 to the basolateral medium. At a dose of 15 μg ml$^{-1}$ of mAb 528, [$^3$H]-thymidine incorporation decreased 73% (Figure 1E; $P$ = 0.01) in HCA-7 cells and 57% (Figure 1F; $P$ = 0.02) in Caco-2 cells. The control antibody UPC-10 did not influence incorporation of [$^3$H]-thymidine (data included in Figure 5C). Thus, DNA replication was affected only when TGF-α or mAb 528 was added to the basolateral medium of polarized HCA-7 and Caco-2 cells, and no significant mitogenic effects were observed when these agents were added to the apical medium or to confluent cells cultured on plastic.

### Qualitative differences in EGFR affinity between polarized and monolayer cultured HCA-7 and Caco-2 cells

In order to further explore the observed differences in DNA synthesis between these cells cultured as a monolayer on plastic compared to a polarized monolayer on Transwell filters, we examined the EGF binding characteristics of the cells. HCA-7 and Caco-2 cells cultured as a monolayer on plastic demonstrated curvilinear Scatchard plots. In HCA-7 cells, there were 2500 high-affinity EGFRs per cell ($K_{D1}$ = 5 nM) and 16 000 EGFRs with a lower affinity ($K_{D2}$ = 83 nM) (Figure 2A). Caco-2 cells exhibited 1900 high-affinity EGFRs per cell ($K_{D1}$ = 3 nM) and 50 000 EGFRs with a lower affinity ($K_{D2}$ = 13 nM) (Figure 2B). Scatchard analyses of EGF binding to basolateral surfaces of polarized HCA-7 and Caco-2 cells cultured on Transwell filters for 7–10 days post-confluence demonstrated linear Scatchard plots for basolateral EGFRs with 27 000 receptors/cell ($K_D$ = 2.1 nM) in HCA-7 cells (Figure 2C) and 57 000 receptors/cell ($K_D$ = 1 nM) in Caco-2 cells (Figure 2D). The Scatchard plot for apical EGFRs on cells cultured on Transwell filters was curvilinear and similar to the plots for EGFRs on cells cultured on plastic (Figure 2C, D). In the apical compartment of polarized HCA-7 cells, there were 1800 high-affinity EGFRs per cell ($K_{D1}$ = 1.3 nM) and 6000 EGFRs with a lower affinity ($K_{D2}$ = 12 nM) (Figure 2C, inset). In the apical compartment of Caco-2 cells, 550 high-affinity EGFRs per cell ($K_{D1}$ = 1.1 nM) and 10 000 EGFRs with a lower affinity ($K_{D2}$ = 58 nM) (Figure 2D, inset) were observed. The $K_D$ values derived from the higher affinity population of EGFRs of HCA-7 and Caco-2 cells cultured on plastic and from the apical compartment of cells cultured on Transwell filters generally approached the $K_D$ for the EGFRs on the basolateral surface of polarized cells.

### Steady-state mRNA expression of EGFR and its ligands in HCA-7 and Caco-2 cells

The ability of the EGFR mAb 528 to decrease DNA replication led us to consider that endogenous EGFR ligands may regulate baseline mitogenesis in these cells. To pursue this line of investigation, steady-state mRNA expression of EGFR ligands was examined in HCA-7 and Caco-2 cells by Northern blot analysis. Poly-A mRNA was isolated from these cells cultured as a confluent monolayer on plastic and as a polarizing monolayer on Transwell filters under serum-containing conditions and after serum starvation for 48 h. Ten and 6 kb EGFR transcripts (Figure 3A) were identified in both cell lines, and these transcripts appeared stronger under serum-free conditions. A 1.4 kb AR transcript was detected in both cell lines and also appeared stronger under serum-free conditions (Figure 3A). A less intense, 2.5 kb HB-EGF transcript was detected but no transcripts were detected for TGF-α, EGF or BTC under these experimental conditions (data not shown). The lack of TGF-α mRNA was further documented by absence of TGF-α protein in the conditioned medium and cell lysates from both HCA-7 and Caco-2 cells using a highly sensitive and specific radioimmunoassay (Halter et al, 1992) (data not shown). Bishop et al (1994) have previously shown that Caco-2 cells express TGF-α, but in our Caco-2 cells no protein or mRNA could be detected. To further examine EGFR binding ligands in Caco-2 cells, we performed RT-PCR of all the EGFR-binding ligands. Using this approach, an intense AR transcript, and to a lesser extent HB-EGF and TGF-α were detected. Faint, but specific, bands for EGF and BTC mRNA were also detected (Figure 3B).

### Immunolocalization of AR to the basolateral compartment of polarized colon carcinoma cells

Cell surface localization of AR was performed in polarized HCA-7 and Caco-2 cells by immunofluorescence and laser scanning confocal microscopy. We examined AR expression in cells on Transwell filters as they grew as non-polarized single cells until they became confluent and fully polarized. In non-polarized cells, AR was evenly distributed over the cell surface, a finding that was particularly apparent in cells at the leading edge of a monolayer of

© 1999 Cancer Research Campaign

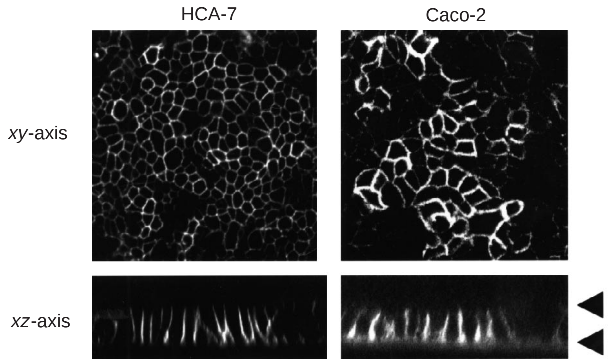

**Figure 4** AR is localized to the basolateral membrane domain in polarized HCA-7 and Caco-2 cells. HCA-7 and Caco-2 cells grown on Transwell filters were fixed and incubated with AR mAb added to both apical and basal compartments. Bound antibodies were visualized by CY-3 conjugated donkey anti-mouse IgG using confocal microscopy. Top portion of each panel represents the *xy*-axis while the bottom portion represents the *xz*-axis analysis. In the *xz*-axis, arrowheads indicate the extent of the basolateral domain small colonies (data not shown). However, AR was localized predominantly to the basolateral compartment in polarized cells from larger colonies and confluent monolayers (Figure 4).

### AR is an autocrine growth factor for polarized HCA-7 and Caco-2 cells

Sequential ectodomain cleavage of AR precursor is responsible for the multiple cellular and soluble AR forms produced by HCA-7 and Caco-2 cells (Brown et al, 1998). To determine whether AR is released in a polarized manner from HCA-7 and Caco-2 cells, cells grown on Transwells were metabolically labelled overnight. Conditioned medium from the apical and basolateral compartments were collected, immunoprecipitated with AR mAb and then analysed by SDS-PAGE and fluorography (Figure 5A). In both cell lines, we detected a major AR species ~43 kDa band in the basolateral conditioned medium. After a 2 h chase, a minor AR doublet of 19 and 21 kDa was also detected (Figure 5B). The molecular weights of the different soluble AR forms released by HCA-7 and Caco-2 cells are in agreement with our previous findings (Brown et al, 1998). Only very low levels of AR species were detected in the apical-conditioned medium. Quantification of AR indicates that > 98% is released into the basolateral-conditioned medium, demonstrating that in HCA-7 and Caco-2 cells AR is released in a polarized manner.

In polarized HCA-7 and Caco-2 cells, the ability of EGFR neutralizing antibody to significantly decrease DNA replication suggested that an endogenous autocrine growth factor is present. AR is a likely candidate because of its abundant expression as well as its localization to, and release from, the basolateral surface, which would allow direct access to basolateral EGFRs. To determine whether endogenous AR acts as an autocrine growth factor under these basal culture conditions, we tested the ability of a cocktail of neutralizing AR antibodies to block mitogenesis of HCA-7 and Caco-2 cells grown as polarized monolayers on Transwell filters. Basolateral administration of neutralizing AR antibodies significantly reduced DNA replication, indicating that AR acts as an autocrine growth factor for these polarized cells (Figure 5C).

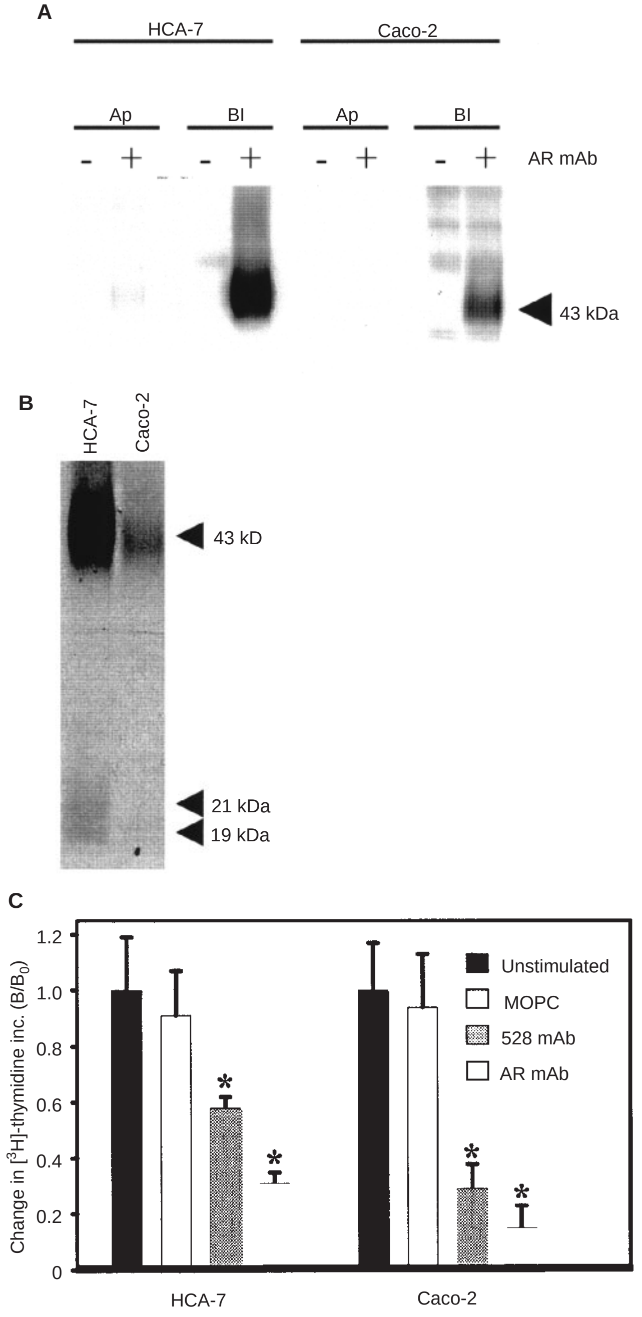

**Figure 5** AR is an autocrine growth factor for polarized HCA-7 and Caco-2 cells. (**A**) AR is released into the basolateral medium of polarized HCA-7 and Caco-2 cells. HCA-7 and Caco-2 cells cultured on Transwell filters were metabolically labelled with [$^{35}$S]-Translabel for 16–20 h. After labelling, apical and basolateral conditioned medium was immunoprecipitated with AR antibody and analysed by SDS-PAGE and fluorography. Apical and basolateral domains are represented by Ap and B1. The position of the major AR species is indicated by the arrowhead and size indicated in kD. In (**B**) HCA-7 and Caco-2 cells were metabolically labeled with [$^{35}$S]-Translabel for 2 h and chased for 2 h. Conditioned basal medium was immunoprecipitated with AR antibody and analysed by SDS-PAGE and fluorography. Position and size of AR species are indicated on the left. (**C**) AR neutralizing antibody decreases basal mitogenesis in polarized HCA-7 and Caco-2 cells. Cells cultured on Transwell filters (7–10 days post-confluence) were serum starved for 48 h. AR neutralizing antibody cocktail, mAb 528 or control antibody (MOPC 21) were administered to the cells for 21 h and [$^{3}$H]-thymidine labelling was performed as described in Materials and Methods. HCA-7 and Caco-2 cells were treated with AR antibody cocktail (32 μg ml$^{-1}$), mAb 528 (3 μg ml$^{-1}$) and MOPC-21 (32 μg ml$^{-1}$) administered to the basolateral compartment. Vertical bars represent standard deviations. *: significant alteration in mitogenic effect compared to baseline values ($P < 0.05$)

### DISCUSSION

Epithelial cells cultured in vitro on plastic as a monolayer do not conform to the in vivo polarized nature of these cells with regard to the spatial compartmentalization of growth factors and their cognate receptors. The ability to culture selected epithelial cell types as a polarized monolayer on a permeable support allows a better representation of in vivo conditions. We have studied the delivery and processing of TGF-α and EGF in polarized MDCK-II and $LLCPK_1$ cells stably transfected with the respective full-length human cDNAs (Dempsey et al, 1994, 1997). TGF-α is delivered directly to the basolateral surface of these polarized cells, where it is cleaved and consumed rapidly by basolateral EGFRs (Dempsey et al, 1994). In contrast to TGF-α, EGF immunoreactivity is found predominantly at the apical surface. However, the EGF precursor is not sorted, but is preferentially cleaved from the basolateral surface to release as a high molecular weight 170 kDa form into the medium that does not interact with basolateral EGFRs (Dempsey et al, 1997). The differential sorting, processing and receptor utilization of TGF-α and EGF underscore the importance of examining peptide growth factor–receptor interactions in epithelial cells in the more physiologically relevant polarized state.

Culturing polarizing epithelial cells on these permeable supports also permits selective access to the apical and/or basolateral compartments. The initial focus of the present studies was to examine DNA replication in two human colon cancer lines that polarize when cultured on Transwell filters and compare the results to cells cultured as a monolayer on plastic. Intriguing differences in DNA synthesis were observed in response to exogenous TGF-α or mAb 528 in HCA-7 and Caco-2 cells under these two different growth conditions. In both cell lines, administration of these agents to confluent cells cultured as a monolayer on plastic or to the apical compartment of polarized cells did not alter DNA synthesis over baseline values. However, basolateral administration of TGF-α and mAb 528 did alter DNA synthesis. There was a dose-dependent increase in proliferation after basolateral administration of TGF-α, which in HCA-7 cells was 8.4-fold and in Caco-2 cells was 3.0-fold. Under these conditions, we also found a dose-dependent decrease in DNA synthesis after EGFR blockade with mAb 528. In HCA-7 cells, the decrease was 73% and in Caco-2 cells was 57%. The difference between the two cell lines is unclear, as Caco-2 cells have a higher number of EGFRs than HCA-7 cells, but the affinity for EGF binding was similar.

There are a number of possible explanations for the proliferative differences between cells cultured on plastic and on Transwell filters. First, there is the question of access. Does exogenous TGF-α or mAb 528 reach basolateral EGFRs in HCA-7 and Caco-2 cells cultured on plastic? Fuller and Simons have reported that there were approximately 32 000 low-affinity transferrin receptors in MDCK cells cultured on plastic. An additional 35 000 high-affinity transferrin receptors were detected after EGTA treatment (Fuller et al, 1986). These authors, and others (Martinez-Palomo et al, 1980; Pesonen et al, 1983), have suggested that EGTA 'opens' the tight junctions and thus uncovers basolateral high-affinity transferrin receptors. Although we were able to bind radiolabelled EGF to the EGFR in both HCA-7 and Caco-2 cells cultured as a monolayer on plastic, it is not known whether the receptor concentration thus obtained was the true value, or represented only a portion of the total receptor population.

Another possible explanation for the differences in mitogenic response is based on the affinity and number of high-affinity EGFRs. Scatchard analysis demonstrated that EGFR affinity and number of high-affinity receptors was higher in the basolateral compartment than in cells cultured on plastic. The majority of EGFRs on HCA-7 and Caco-2 cells cultured as a monolayer on plastic were low-affinity, whereas all the basolateral EGFRs in cells cultured on Transwell filters were high-affinity. The fact that the EGFR Scatchard plots for cells cultured on plastic were curvilinear suggests that ternary complexes were formed by the receptor, ligand and another protein(s) (Gex-Fabry et al, 1986; Mayo et al, 1989; Wofsy et al, 1992). There are a number of observations that favour the correlation between EGFR affinity and biological effect (Defize et al, 1989; Bellot et al, 1990). Others have found a similar correlation between the affinity state of the EGFR and proliferative effect after growth factor administration (Fowler et al, 1995). It has been shown that a T → G point mutation in the EGFR at residue 743 results in the *wa*-2 phenotype (Luetteke et al, 1994; Fowler et al, 1995), which is characterized by loss of high-affinity EGFRs and a requirement for a 20-fold higher concentration of EGF to achieve the same effect on DNA replication as seen with the cells bearing wild-type EGFRs (Fowler et al, 1995).

The ability of EGFR blockade to attenuate mitogenesis prompted us to consider whether endogenous EGFR ligand(s) contribute to baseline DNA replication. A number of reports have documented the presence of EGFRs in Caco-2 cells (Auricchio et al, 1994; Bishop et al, 1994; Milovic et al, 1995), and the presence of autocrine growth factor(s) operating through the EGFR has been hypothesized (Bishop et al, 1994). We found that HCA-7 and Caco-2 cells express AR and, to a much lesser extent, HB-EGF. Expression for TGF-α, EGF or BTC was not detected by Northern blot analysis. Furthermore, TGF-α protein was not detected in the conditioned medium or cell lysate using a sensitive and specific TGF-α radioimmunoassay (Halter et al, 1992). However, we detected TGF-α mRMA by RT-RCR, which is in agreement with the results by Bishop et al (1994). Therefore, TGF-α is below the detection limit of our radioimmunoassay and Northern blot analysis.

We have previously found that mRNA expression for AR is more uniformly increased in human colon carcinomas than adjacent normal mucosa compared with relative TGF-α expression in colon cancers and normal tissues (Cook et al, 1992). In the human colon carcinoma cell line (Geo) cultured as a monolayer on plastic, removal of AR from the conditioned medium by AR antibody immunoprecipitation, resulted in a 40% reduction in proliferation (Johnson et al, 1992), indicating that AR acts as an autocrine growth factor for these cells under these culture conditions. Herein, stringent criteria qualify AR as an autocrine growth factor for polarized HCA-7 and Caco-2 cells: it is produced and delivered to the 'EGFR-bearing' basolateral surface of these polarized cells, it is released into the basolateral medium, and antibody neutralization of AR in this basolateral compartment attenuates baseline mitogenesis.

In summary, we demonstrate that AR is the predominant EGF-like ligand produced by HCA-7 and Caco-2 cells, and that it appears to regulate growth in an autocrine manner. Exogenous TGF-α or an EGFR neutralizing antibody affects mitogenesis of polarized HCA-7 and Caco-2 cells, but only when these agents are added to the basolateral medium. Thus, the spatial organization of epithelial cells may be necessary for the mitogenic effects of EGF-like family members, and this may, in part, be due to increased EGFR affinity and number of high-affinity EGFRs in the basolateral compartment of polarized epithelial cells.

 *© 1999 Cancer Research Campaign*

### ACKNOWLEDGEMENTS

Grant support: this work was supported by the Danish Cancer Society, Grant 93-031 to L Damstrup, by National Institute of Health Grant CA46413 to RJ Coffey Jr, ACS grant IRG178E to SK Kuwada and grant DAMD 17-95-5-4444 to HS Wiley. SK Kuwada is supported by the office of research and development department of Veterans Affairs. RJ Coffey Jr is a Veterans Association Clinical Investigator. The authors acknowledge the generous support of the Peter Powell Memorial Fund. Laser scanning confocal microscopy was performed in the Vanderbilt University Medical Center Cell Imaging Resource (supported by CA68485 and DK20593). We also thank those mentioned under Materials and Methods for generously supplying reagents.

### REFERENCES

Amemiya K, Kurachi H, Adachi H, Morishige KI, Adachi K, Imai T and Miyake A (1994) Involvement of epidermal growth factor (EGF)/EGF receptor autocrine and paracrine mechanism in human trophoblast cells: functional differentiation in vitro. *J Endocrinol* **143**: 291–301

Auricchio A, Di Domenico M, Castoria G, Bilancio A and Migliaccio A (1994) Epidermal growth factor induces protein tyrosine phosphorylation and association of p190 with ras-GTP-ase activating protein in Caco-2 cells. *FEBS Lett* **353**: 16–20

Basson MD, Beidler DR, Turowski G, Zarif A, Modlin IM, Jena BP and Madri JA (1994) Effect of tyrosine kinase inhibition on basal and epidermal growth factor-stimulated human Caco-2 enterocyte sheet migration and proliferation. *J Cell Physiol* **160**: 491–501

Basson MD, Modlin IM and Madri JA (1992). Human enterocyte (Caco-2) migration is modulated in vitro by extracellular matrix composition and epidermal growth factor. *J Clin Invest* **90**: 15–23

Bellot F, Moolenaar W, Kris R, Mirakhur B, Verlaan I, Ullrich A, Schlessinger J and Felder S (1990) High-affinity epidermal growth factor binding is specifically reduced by a monoclonal antibody, and appears necessary for early responses. *J Cell Biol* **110**: 491–502

Bishop WP and Wen JT (1994) Regulation of Caco-2 cell proliferation by basolateral membrane epidermal growth factor receptors. *Am J Physiol* **267**: G892–G900

Brown CL, Meise KS, Plowman GD, Coffey RJ and Dempsey PJ (1998) Cell surface ectodomain cleavage of human amphiregulin precursor is sensitive to a metalloprotease inhibitor. Release of a predominant N-glycosylated 43-kDa soluble form. *J Biol Chem* **273**: 17258–17268

Chen P, Gupta K and Wells A (1994) Cell movement elicited by epidermal growth factor receptor requires kinase and autophosphorylation but is separable from mitogenesis. *J Cell Biol* **124**: 547–555

Chinery R and Cox J (1995) Modulation of epidermal growth factor effects on epithelial ion transport by intestinal trefoil factor. *Br J Pharmacol* **115**: 77–80

Coffey RJ, Hawkey C, Damstrup L, Graves-Deal R, Daniel VC, Dempsey PJ, Chinery R, Kirkland S, DuBois RN, Jetton TL and Morrow JD (1997) Vectorial release and TGFα mediated production of prostaglanding is polarized colon carcinoma cell lines. *Proc Natl Acad Sci USA* **94**: 657–662

Cook PW, Pittelkow MR, Keeble WW, Graves-Deal R, Coffey RJ Jr and Shipley GD (1992) Amphiregulin messenger RNA is elevated in psoriatic epidermis and gastrointestinal carcinomas. *Cancer Res* **52**: 3224–3227

Culouscou J-M, Remacle-Bonnet M, Carlton GW, Plowman GD and Shoyab M (1992) Colorectum cell-derived growth factor (CRDGF) is homologous to amphiregulin, a member of the epidermal growth factor family. *Growth Factors* **7**: 195–205

Danielson PE, Forss-Petter S, Brow MA, Calavetta L, Douglass J, Milner RJ and Sutcliffe JG (1988) p1B15: a cDNA clone of the rat mRNA encoding cyclophilin. DNA **7**: 261–267

Defize LH, Boonstra J, Meisenhelder J, Kruijer W, Tertoolen LG, Tilly BC, Hunter T, Van Bergen En Henegouwen PM, Moolenaar WH and De Laat SW (1989) Signal transduction by epidermal growth factor occurs through the subclass of high affinity receptors. *J Cell Biol* **109**: 2495–2507

Dempsey PJ and Coffey RJ, Jr (1994) Basolateral targeting and efficient consumption of transforming growth factor-α when expressed in Madin-Darby canine kidney cells. *J Biol Chem* **269**: 16878–16889

Dempsey PJ, Meise K, Yoshitake Y, Nishikawa K and Coffey RJ (1997) Apical enrichment of human EGF presursor in Madin-Darby canine kidney cells involves preferential basolateral ectodomain cleavage sensitive to a metalloprotease inhibitor. *J Cell Biol* **138**: 1–12

Dobner PR, Kawasaki ES, Yu LY and Bancroft FC (1981) Thyroid or glucocorticoid hormone induces pre-growth-hormone mRNA and its probable nuclear precursor in rat pituitary cells. *Proc Natl Acad Sci USA* **78**: 2230–2234

Feldman HA (1972) Mathematical theory of complex ligand-binding systems at equilibrium: some methods for parameter fitting. *Analyt Biochem* **48**: 317–338

Fowler KJ, Walker F, Alexander W, Hibbs ML, Nice EC, Bohmer RM, Mann GB, Thumwood C, Maglitto R, Danks JA, Chetty R, Burgess AW and Dunn AR (1995) A mutation in the epidermal growth factor receptor in waved-2 mice has a profound effect on receptor biochemistry that results in impaired lactation. *Proc Natl Acad Sci USA* **92**: 1465–1469

Fuller SD and Simons K (1986) Transferrin receptor polarity and recycling accuracy in “tight” and “leaky” strains of Madin-Darby canine kidney cells. *J Cell Biol* **103**: 1767–1779

Gex-Fabry M and Delisi C (1986) Regulation of interacting populations during endocytosis: models of growth factor-tumor promoter dynamics. *Am J Physiol* **250**: R1123–R1132

Halter SA, Dempsey PJ, Matsui Y, Stokes MK, Graves-Deal R, Hogan BLM and Coffey RJ Jr (1992) Distinctive patterns of hyperplasia in transgenic mice with mouse mammary tumor virus transforming growth factor-α. Characterization of mammary gland and skin proliferation. *Am J Pathol* **140**: 1131–1146

Hobert M and Carlin C (1995) Cytoplasmic juxtamembrane domain of the human EGF receptor is required for basolateral localization in MDCK cells. *J Cell Physiol* **162**: 434–446

Johnson GR, Saeki T, Gordon AW, Shoyab M, Salomon DS and Stromberg K (1992) Autocrine action of amphiregulin in a colon carcinoma cell line and immunocytochemical localization of amphiregulin in human colon. *J Cell Biol* **118**: 741–751

Kirkland SC (1985) Dome formation by a Human Colonic Adenocarcinoma cell line (HCA-7). *Cancer Res* **45**: 3790–3795

Lebivic G, Quaroni A and Rodriguez-Boulan E (1991) Microtubular organization and its involvement in the biogenetic pathways of plasma membrane proteins in Caco-2 intestinal epithelial cells. *J Cell Biol* **113**: 275–288

Li S, Plowman GD, Buckley SD and Shipley GD (1992). Heparin inhibition of autonomous growth implicates amphiregulin as an autocrine growth factor for normal human mammary epithelial cells. *J Cell Physiol* **153**: 103–111

Luetteke NC, Phillips HK, Qiu TH, Copeland NG, Earp HS, Jenkins NA and Lee DC (1994) The mouse waved-2 phenotype results from a point mutation in the EGF receptor tyrosine kinase. *Genes Dev* **8**: 399–413

Maratos-Flier E, Kao C-YY, Verdin EM and King GL (1987) Reseptor-mediated vectorial transcytosis of epidermal growth factor by Madin-Darby canine kidney cells. *J Cell Biol* **105**: 1595–1601.

Martinez-Palomo A, Meza I, Beaty G and Cereijido M (1980) Experimental modulation of occluding junctions in a cultured transporting epithelium. *J Cell Biol* **87**: 736–745.

Mayo KH, Nunez M, Burke C, Starbuck C, Lauffenburger D and Savage CR, Jr (1989) Epidermal growth factor receptor binding is not a simple one-step process. *J Biol Chem* **264**: 17838–17844

Melton DA, Krieg PA, Rebagliati MR, Maniatis T, Zinn K and Green MR (1984) Efficient in vitro synthesis of biologically active RNA and RNA hybridization probes from plasmids containing a bacteriophage SP6 promoter. *Nucleic Acids Res* **12**: 7035–7056

Milovic V, Deubner C, Zeuzem S, Piiper A, Caspary WF and Stein J (1995) EGF stimulates polyamine uptake in Caco-2 cells. *Biochem Biophys Res Commun* **206**: 962–968

Pesonen M and Simons K (1983) Transepithelial transport of a viral membrane glycoprotein implanted into the apical plasma membrane of Madin-Darby canine kidney cells. II. Immunological quantitation. *J Cell Biol* **97**: 638–643

Qi C-F, Liscia DS, Normanno N, Merlo G, Johnson GR, Gullick WJ, Ciardiello F, Saeki T, Brandt R, Kim N, Kenney N and Solomon DS (1994). Expression of transforming growth factor alpha, amphiregulin and cripto-1 in human breast carcinomas. *Br J Cancer* **69**: 903–910

Scatchard G (1949) The attractions of proteins for small molecules and ions. *Ann NY Acad Sci* **51**: 660–672

Traverse S, Seedorf K, Paterson H, Marshall CJ, Cohen P and Ullrich A (1994) EGF triggers neuronal differentiation of PC12 cells that overexpress the EGF receptor. *Curr Biol* **4**: 694–701

Wofsy C, Goldstein B, Lund K and Wiley HS (1992). Implications of epidermal growth factor (EGF) induced EGF receptor aggregation. *Biophys J* **63**: 98–110

Ziober BL, Willson JK, Hymphrey LE, Childress-Fields K and Brattain MG (1993) Autocrine transforming growth factor-alpha is associated with progression of transformed properties in human colon cancer cells. *J Biol Chem* **268**: 691–698

© 1999 Cancer Research Campaign

## References

[PDF_00816] Amemiya K., Kurachi H., Adachi H., Morishige K. I., Adachi K., Imai T., Miyake A. (1994). Involvement of epidermal growth factor (EGF)/EGF receptor autocrine and paracrine mechanism in human trophoblast cells: functional differentiation in vitro.. J Endocrinol.

[PDF_00820] Auricchio A., di Domenico M., Castoria G., Bilancio A., Migliaccio A. (1994). Epidermal growth factor induces protein tyrosine phosphorylation and association of p190 with ras-GTP-ase activating protein in Caco-2 cells.. FEBS Lett.

[PDF_00868] Bannykh S. I., Balch W. E. (1997). Membrane dynamics at the endoplasmic reticulum-Golgi interface.. J Cell Biol.

[PDF_00824] Basson M. D., Beidler D. R., Turowski G., Zarif A., Modlin I. M., Jena B. P., Madri J. A. (1994). Effect of tyrosine kinase inhibition on basal and epidermal growth factor-stimulated human Caco-2 enterocyte sheet migration and proliferation.. J Cell Physiol.

[PDF_00828] Basson M. D., Modlin I. M., Madri J. A. (1992). Human enterocyte (Caco-2) migration is modulated in vitro by extracellular matrix composition and epidermal growth factor.. J Clin Invest.

[PDF_00831] Bellot F., Moolenaar W., Kris R., Mirakhur B., Verlaan I., Ullrich A., Schlessinger J., Felder S. (1990). High-affinity epidermal growth factor binding is specifically reduced by a monoclonal antibody, and appears necessary for early responses.. J Cell Biol.

[PDF_00835] Bishop W. P., Wen J. T. (1994). Regulation of Caco-2 cell proliferation by basolateral membrane epidermal growth factor receptors.. Am J Physiol.

[PDF_00838] Brown C. L., Meise K. S., Plowman G. D., Coffey R. J., Dempsey P. J. (1998). Cell surface ectodomain cleavage of human amphiregulin precursor is sensitive to a metalloprotease inhibitor. Release of a predominant N-glycosylated 43-kDa soluble form.. J Biol Chem.

[PDF_00842] Chen P., Gupta K., Wells A. (1994). Cell movement elicited by epidermal growth factor receptor requires kinase and autophosphorylation but is separable from mitogenesis.. J Cell Biol.

[PDF_00845] Chinery R., Cox H. M. (1995). Modulation of epidermal growth factor effects on epithelial ion transport by intestinal trefoil factor.. Br J Pharmacol.

[PDF_00847] Coffey R. J., Hawkey C. J., Damstrup L., Graves-Deal R., Daniel V. C., Dempsey P. J., Chinery R., Kirkland S. C., DuBois R. N., Jetton T. L. (1997). Epidermal growth factor receptor activation induces nuclear targeting of cyclooxygenase-2, basolateral release of prostaglandins, and mitogenesis in polarizing colon cancer cells.. Proc Natl Acad Sci U S A.

[PDF_00851] Cook P. W., Pittelkow M. R., Keeble W. W., Graves-Deal R., Coffey R. J., Shipley G. D. (1992). Amphiregulin messenger RNA is elevated in psoriatic epidermis and gastrointestinal carcinomas.. Cancer Res.

[PDF_00854] Culouscou J. M., Remacle-Bonnet M., Carlton G. W., Plowman G. D., Shoyab M. (1992). Colorectum cell-derived growth factor (CRDGF) is homologous to amphiregulin, a member of the epidermal growth factor family.. Growth Factors.

[PDF_00858] Danielson P. E., Forss-Petter S., Brow M. A., Calavetta L., Douglass J., Milner R. J., Sutcliffe J. G. (1988). p1B15: a cDNA clone of the rat mRNA encoding cyclophilin.. DNA.

[PDF_00861] Defize L. H., Boonstra J., Meisenhelder J., Kruijer W., Tertoolen L. G., Tilly B. C., Hunter T., van Bergen en Henegouwen P. M., Moolenaar W. H., de Laat S. W. (1989). Signal transduction by epidermal growth factor occurs through the subclass of high affinity receptors.. J Cell Biol.

[PDF_00865] Dempsey P. J., Coffey R. J. (1994). Basolateral targeting and efficient consumption of transforming growth factor-alpha when expressed in Madin-Darby canine kidney cells.. J Biol Chem.

[PDF_00872] Dobner P. R., Kawasaki E. S., Yu L. Y., Bancroft F. C. (1981). Thyroid or glucocorticoid hormone induces pre-growth-hormone mRNA and its probable nuclear precursor in rat pituitary cells.. Proc Natl Acad Sci U S A.

[PDF_00875] Feldman H. A. (1972). Mathematical theory of complex ligand-binding systems of equilibrium: some methods for parameter fitting.. Anal Biochem.

[PDF_00877] Fowler K. J., Walker F., Alexander W., Hibbs M. L., Nice E. C., Bohmer R. M., Mann G. B., Thumwood C., Maglitto R., Danks J. A. (1995). A mutation in the epidermal growth factor receptor in waved-2 mice has a profound effect on receptor biochemistry that results in impaired lactation.. Proc Natl Acad Sci U S A.

[PDF_00882] Fuller S. D., Simons K. (1986). Transferrin receptor polarity and recycling accuracy in "tight" and "leaky" strains of Madin-Darby canine kidney cells.. J Cell Biol.

[PDF_00885] Gex-Fabry M., DeLisi C. (1986). Regulation of interacting populations during endocytosis: models of growth factor-tumor promoter dynamics.. Am J Physiol.

[PDF_00901] Gilbert T., Le Bivic A., Quaroni A., Rodriguez-Boulan E. (1991). Microtubular organization and its involvement in the biogenetic pathways of plasma membrane proteins in Caco-2 intestinal epithelial cells.. J Cell Biol.

[PDF_00888] Halter S. A., Dempsey P., Matsui Y., Stokes M. K., Graves-Deal R., Hogan B. L., Coffey R. J. (1992). Distinctive patterns of hyperplasia in transgenic mice with mouse mammary tumor virus transforming growth factor-alpha. Characterization of mammary gland and skin proliferations.. Am J Pathol.

[PDF_00892] Hobert M., Carlin C. (1995). Cytoplasmic juxtamembrane domain of the human EGF receptor is required for basolateral localization in MDCK cells.. J Cell Physiol.

[PDF_00895] Johnson G. R., Saeki T., Gordon A. W., Shoyab M., Salomon D. S., Stromberg K. (1992). Autocrine action of amphiregulin in a colon carcinoma cell line and immunocytochemical localization of amphiregulin in human colon.. J Cell Biol.

[PDF_00899] Kirkland S. C. (1985). Dome formation by a human colonic adenocarcinoma cell line (HCA-7).. Cancer Res.

[PDF_00904] Li S., Plowman G. D., Buckley S. D., Shipley G. D. (1992). Heparin inhibition of autonomous growth implicates amphiregulin as an autocrine growth factor for normal human mammary epithelial cells.. J Cell Physiol.

[PDF_00907] Luetteke N. C., Phillips H. K., Qiu T. H., Copeland N. G., Earp H. S., Jenkins N. A., Lee D. C. (1994). The mouse waved-2 phenotype results from a point mutation in the EGF receptor tyrosine kinase.. Genes Dev.

[PDF_00910] Maratos-Flier E., Kao C. Y., Verdin E. M., King G. L. (1987). Receptor-mediated vectorial transcytosis of epidermal growth factor by Madin-Darby canine kidney cells.. J Cell Biol.

[PDF_00913] Martinez-Palomo A., Meza I., Beaty G., Cereijido M. (1980). Experimental modulation of occluding junctions in a cultured transporting epithelium.. J Cell Biol.

[PDF_00916] Mayo K. H., Nunez M., Burke C., Starbuck C., Lauffenburger D., Savage C. R. (1989). Epidermal growth factor receptor binding is not a simple one-step process.. J Biol Chem.

[PDF_00919] Melton D. A., Krieg P. A., Rebagliati M. R., Maniatis T., Zinn K., Green M. R. (1984). Efficient in vitro synthesis of biologically active RNA and RNA hybridization probes from plasmids containing a bacteriophage SP6 promoter.. Nucleic Acids Res.

[PDF_00923] Milovic V., Deubner C., Zeuzem S., Piiper A., Caspary W. F., Stein J. (1995). EGF stimulates polyamine uptake in Caco-2 cells.. Biochem Biophys Res Commun.

[PDF_00926] Pesonen M., Simons K. (1983). Transepithelial transport of a viral membrane glycoprotein implanted into the apical plasma membrane of Madin-Darby canine kidney cells. II. Immunological quantitation.. J Cell Biol.

[PDF_00929] Qi C. F., Liscia D. S., Normanno N., Merlo G., Johnson G. R., Gullick W. J., Ciardiello F., Saeki T., Brandt R., Kim N. (1994). Expression of transforming growth factor alpha, amphiregulin and cripto-1 in human breast carcinomas.. Br J Cancer.

[PDF_00935] Traverse S., Seedorf K., Paterson H., Marshall C. J., Cohen P., Ullrich A. (1994). EGF triggers neuronal differentiation of PC12 cells that overexpress the EGF receptor.. Curr Biol.

[PDF_00938] Wofsy C., Goldstein B., Lund K., Wiley H. S. (1992). Implications of epidermal growth factor (EGF) induced egf receptor aggregation.. Biophys J.

[PDF_00940] Ziober B. L., Willson J. K., Hymphrey L. E., Childress-Fields K., Brattain M. G. (1993). Autocrine transforming growth factor-alpha is associated with progression of transformed properties in human colon cancer cells.. J Biol Chem.

